# IMPACT OF THE MULTI-COMPONENT HOME-BASED PHYSICAL THERAPY INTERVENTION ON COGNITIVE OUTCOMES IN THE CAP TRIAL

**DOI:** 10.1093/geroni/igz038.1567

**Published:** 2019-11-08

**Authors:** Ann Gruber-Baldini

**Affiliations:** University of Maryland Baltimore School of Medicine, Baltimore, Maryland, United States

## Abstract

Cognitive impairment after hip fracture influences recovery and some RCTs suggest aerobic and resistance exercise may improve cognition. This presentation examines differences in PUSH versus PULSE on cognition and the impact of cognition on community ambulation. In CAP, the Modified Mini-Mental State Examination (3MS) was pre-specified for subgroup (3MS<91 versus 3MS≥91) evaluation and as a tertiary outcome. The CAP Mechanistic Pathways ancillary study (subgroup n=40) included Hooper Visual Organization Test and Trails A&B as additional outcomes. At baseline, PUSH did not differ from PULSE on cognitive measures. Over 16 weeks, PUSH became faster on Trails A (Δ= 6.7(29.9)) while PULSE became slower (Δ=9.4(24.2)) (p<.05). Those with higher baseline cognition were more likely to become community ambulators (28.3% 3MS≥91 versus 8.8% 3MS<91), but PUSH versus PULSE effects didn’t differ by 3MS (no effect modification). Results suggest potential differences in recovery by cognition, but no treatment effect differences by level of cognition.

